# Touching with the eyes: Oculomotor self-touch induces illusory body ownership

**DOI:** 10.1016/j.isci.2023.106708

**Published:** 2023-04-26

**Authors:** Antonio Cataldo, Massimiliano Di Luca, Ophelia Deroy, Vincent Hayward

## Main text

(iScience *26*, 106180; March 17, 2023)

The corresponding author footnotes in the original article did not include Ophelia Deroy as a corresponding author. We have now added O.D. as a corresponding author due to her role in providing resources and supervision for the study and her specialized contribution to the philosophical implications of our results. The change does not affect any of the other sections in the article or its conclusions. The authors apologize for any confusion caused to the readers.

